# Supplementary material for: Persistent Elevation in Urinary Neutrophil Gelatinase-Associated Lipocalin Levels Can Be a Predictor of Urinary Tract Infection Recurrence or Persistence in Women
Source: Int J Mol Sci. 2024 Nov 26;25(23):12670. doi: 10.3390/ijms252312670 (PMC11641091; doi:10.3390/ijms252312670)
Supplement: Supplementary file 1 [file ijms-25-12670-s001.zip › ijms-3277863-supplementary.pdf]

International Journal of
Molecular Sciences

MDPI

Supplement Table S1. Baseline urinary cytokine levels between controls and patients with a history of recurrent urinary tract infection and different urinary leukocyte counts

Urine biomarker	Control (n=7)	WBC≦10 (n=12)	WBC 10-75 (n=19)	WBC≧75 (n=14)	p-value
NGAL	32.18±45.96	38.7±39.3	305 ± 472	670 ± 540	<0.001
NGF	0.13±0.01	0.12±0.01	0.15±0.06	0.13±0.03	0.458
CXCL 1	0.95±0.7	0.58±0.21	77.84±238.62	117.99±155.88	0.245
IL-8	5.21±6.57	3.77±4.57	444.48±1546.9	180.26±144.18	0.564
CXCL 10	12.34±23	2.45±2.68	279.45±890.37	507.16±1252.89	0.422
MCP1	148.8±209.76	43.09±41.12	227.99±448.08	296.71±270.12	0.224
TNFα	1.22±0.14	1.14±0.12	6.26±13.14	7.71±9.83	0.226

*comparison between patients of recurrent urinary tract infection with baseline WBC ≤ 10/HPF and WBC >10/HPF. NGAL: neutrophil gelatinase-associated lipocalin, NGF: nerve growth factor, CXCL1: CXC-motif chemokine ligand-1, IL-8: interleukin-8, MCP-1: monocyte chemoattractant protein-1, TNF-α: tumor necrosis factor- alpha; WBC: white blood cell count per high power field; NGAL unit: ng/ml, cytokine unit: pg/ml.

Supplement Table S2. The baseline urine cytokines between controls and patients <u>with a history of recurrent urinary tract infection</u>

	Time	Patient number#	Control (n=7)	Recurrent UTI (n=40)	p-value @
**NGAL**	Baseline	40	32.2 ± 46.0	340 ± 440	0.073
	1 week	37		287 ± 604	
	1 month	40		267 ± 649	
	3 month	38		165 ± 427*	
**NGF**	Baseline	40	0.13 ± 0.01	0.14 ± 0.04	0.584
	1 week	37		0.15 ± 0.06	
	1 month	40		0.11 ± 0.06*	
	3 month	38		0.11 ± 0.05	
**CXCL-1**	Baseline	40	0.95 ± 0.7	78.1 ± 190	0.293
	1 week	37		118 ± 364	
	1 month	40		52.3 ± 167	
	3 month	38		32.2 ± 88.1	
**IL-8**	Baseline	40	5.21 ± 6.57	267 ± 1069	0.525
	1 week	37		306 ± 1390	
	1 month	40		226 ± 1138	
	3 month	38		24.5 ± 42.2*	
**CXCL-10**	Baseline	40	12.3 ± 23.0	310 ± 960	0.420
	1 week	37		147 ± 816	
	1 month	40		56.6 ± 309	
	3 month	38		57.6 ± 305	
**MCP-1**	Baseline	40	149 ± 210	209 ± 353	0.663
	1 week	37		263 ± 667	
	1 month	40		159 ± 332	
	3 month	38		183 ± 332	
**TNF-α**	Baseline	40	1.22 ± 0.14	5.86 ± 10.8	0.266
	1 week	37		6.58 ± 19.4	
	1 month	40		5.13 ± 15.8	
	3 month	38		2.12 ± 3.61*	

# patient number of recurrent urinary tract infection, @ p values among subgroups

* P<0.05 compared with baseline; abbreviations: same as in footnotes of table 1

Unit of urine biomarker: NGAL: ng/ml, other biomarkers: pg/ml

Supplement Table S3. Changes of urine cytokines between patients with and without urinary tract infection persistence or recurrence within 1 month after acute cystitis episode

	Time	UTI persistence or recurrence (n=12)	No UTI persistence or recurrence (n=28)	p-value#
**NGAL**	Baseline	685 ± 657	193 ± 169	0.026
	1 week	803 ± 895	51.0 ± 74.5*	0.019
	1 month	824 ± 1003	28.1 ± 30.8*	0.019
	3 month	527 ± 724	30.1 ± 38.3*	0.058
**NGF**	Baseline	0.15 ± 0.06	0.13 ± 0.03	0.274
	1 week	0.18 ± 0.09	0.13 ± 0.03	0.043
	1 month	0.14 ± 0.11	0.10 ± 0.03*	0.277
	3 month	0.12 ± 0.05	0.11 ± 0.05	0.817
**CXCL-1**	Baseline	204 ± 315	24.2 ± 39.0	0.075
	1 week	367 ± 593	3.48 ± 9.49*	0.069
	1 month	172 ± 277	1.0 ± 1.03*	0.055
	3 month	110 ± 147	3.35 ±10.9*	0.047
**IL-8**	Baseline	697 ± 1932	82.3 ± 115	0.294
	1 week	944 ± 2433	13.7 ± 15.4*	0.233
	1 month	742 ± 2044	4.42 ± 5.18*	0.237
	3 month	55.9 ± 66.6	12.9 ± 20.8*	0.074
**CXCL-10**	Baseline	241 ± 506	340 ± 1106	0.769
	1 week	460 ± 1452	3.96 ± 4.78	0.322
	1 month	182 ± 560	2.99 ± 5.21	0.293
	3 month	202 ± 583	4.18 ± 11.3	0.312
**MCP-1**	Baseline	337 ± 578	155 ± 183	0.306
	1 week	580 ± 1147	117 ± 127	0.210
	1 month	348 ± 535	77.8 ± 142*	0.112
	3 month	322 ± 392	132 ± 298	0.123
**TNF-α**	Baseline	8.96 ± 16.1	4.54 ± 7.6	0.241
	1 week	17.1 ± 33.2	1.77 ± 0.78	0.157
	1 month	13.3 ± 27.9	1.65 ± 0.4	0.178
	3 month	4.46 ± 6.57	1.25 ± 0.51*	0.158

* P<0.05 compared with baseline in the same group; # p values among subgroups

Abbreviations: same as in footnotes of table 1

Unit of urine biomarker: NGAL: ng/ml, other biomarkers: pg/ml

Supplement Table S4. The changes of urinary cytokines among patients with persistent urinary tract infection (UTI), recurrent UTI, and no UTI recurrence

	Time	Persistent UTI (n=9)	Recurrent UTI (n=10)	No UTI recurrence(n=21)	Control (n=7)	p-value#
NGAL	Baseline	641±640	300±389	182±168	32.2 ± 46.0	0.054
	1 week	819±879	70.9±107	23.7±22.4*		0.007
	1M	884±1030	39.1±33.0*	29.2±34.2*		0.010
	3M	508±737	48.9±55.4	30.3±34.0*		0.055
NGF	Baseline	0.15±0.07	0.12±0.03	0.14±0.03	0.13 ± 0.01	0.312
	1 week	0.17±0.09	0.14±0.03	0.13±0.04		0.209
	1M	0.14±0.11	0.09±0.02*	0.11±0.03*		0.219
	3M	0.12±0.05	0.12±0.05	0.11±0.05		0.892
CXCL-1	Baseline	209±329	45.6±68.5	17.9±28.2	0.95 ± 0.7	0.079
	1 week	368±592	5.87±14.6	0.88±0.51*		0.048
	1M	187±286	2.19±3.29	0.92±0.89*		0.038
	3M	1102±147	6.82±17.7	1.42±1.9*		0.031
IL-8	Baseline	729±2021	136±187	63.9±80.7	5.21 ± 6.57	0.243
	1 week	953±2429	16.4±14.7	4.74±4.67*		0.238
	1M	803±2132	11.2±13.7	3.56±4.93*		0.262
	3M	54.0±68.0	19.9±23.4	9.89±18.7*		0.078
CXCL-10	Baseline	385±1154	661±1409	50.4±86.3	12.3 ± 23	0.356
	1 week	462±1452	3.79±5.73	2.81±3.19*		0.313
	1M	199±584	3.6±5.97	2.25±1.67*		0.205
	3M	202±584	7.18±17.8	2.53±4.57*		0.361
MCP-1	Baseline	392±588	167±250	124±125	149 ± 210	0.124
	1 week	629±1125	84.0±113	102±126		0.085
	1M	351±563	119±177	65.9±121		0.149
	3M	308±392	190±482	105±121		0.315
TNF-α	Baseline	9.54±16.8	5.42±10.2	3.89±5.43	1.22 ± 0.14	0.400
	1 week	17.5±33.0	1.44±0.33	1.67±0.39		0.127
	1M	14.2±29.1	1.71±0.59	1.66±0.27		0.179
	3M	4.43±6.58	1.35±0.61	1.21±0.49		0.155

* P<0.05 compared with baseline in the same group; # p values among subgroups

Abbreviations: same as in footnotes of table 1

Unit of urine biomarker: NGAL: ng/ml, other biomarkers: pg/ml

Supplement Table S5

STROBE Statement—Checklist of items that should be included in reports of ***case-control studies***

	Item No	Recommendation	Page No
**Title and abstract**	1	(*a*) Indicate the study's design with a commonly used term in the title or the abstract	L 2-4
		(*b*) Provide in the abstract an informative and balanced summary of what was done and what was found	L 34-39
**Introduction**			
Background/rationale	2	Explain the scientific background and rationale for the investigation being reported	L 45-68
Objectives	3	State specific objectives, including any prespecified hypotheses	L 68-71
**Methods**			
Study design	4	Present key elements of study design early in the paper	L 289-292
Setting	5	Describe the setting, locations, and relevant dates, including periods of recruitment, exposure, follow-up, and data collection	L 294-295
Participants	6	(*a*) Give the eligibility criteria, and the sources and methods of case ascertainment and control selection. Give the rationale for the choice of cases and controls	L 295-299
		(*b*) For matched studies, give matching criteria and the number of controls per case	L 305-308
Variables	7	Clearly define all outcomes, exposures, predictors, potential confounders, and effect modifiers. Give diagnostic criteria, if applicable	L 347-351
Data sources/ measurement	8*	For each variable of interest, give sources of data and details of methods of assessment (measurement). Describe comparability of assessment methods if there is more than one group	L 321-328
Bias	9	Describe any efforts to address potential sources of bias	L 273-278
Study size	10	Explain how the study size was arrived at	L 292-294
Quantitative variables	11	Explain how quantitative variables were handled in the analyses. If applicable, describe which groupings were chosen and why	L 353-356
Statistical methods	12	(*a*) Describe all statistical methods, including those used to control for confounding	L 356-358
		(*b*) Describe any methods used to examine subgroups and interactions	L 351-353
		(*c*) Explain how missing data were addressed	
		(*d*) If applicable, explain how matching of cases and controls was addressed	
		(*e*) Describe any sensitivity analyses	
**Results**			
Participants	13*	(a) Report numbers of individuals at each stage of study—eg numbers potentially eligible, examined for eligibility, confirmed eligible, included in the study, completing follow-up, and analysed	L 73-77
		(b) Give reasons for non-participation at each stage	L 75-77
		(c) Consider use of a flow diagram	
Descriptive data	14*	(a) Give characteristics of study participants (eg demographic, clinical, social) and information on exposures and potential confounders	L 79-82
		(b) Indicate number of participants with missing data for each variable of interest	

Outcome data	15*	Report numbers in each exposure category, or summary measures of exposure	L 79-82

Main results	16	(*a*) Give unadjusted estimates and, if applicable, confounder-adjusted estimates and their precision (eg, 95% confidence interval). Make clear which confounders were adjusted for and why they were included	L 94-
		(*b*) Report category boundaries when continuous variables were categorized	
		(*c*) If relevant, consider translating estimates of relative risk into absolute risk for a meaningful time period	
Other analyses	17	Report other analyses done—eg analyses of subgroups and interactions, and sensitivity analyses	Figure 1-3
**Discussion**			
Key results	18	Summarise key results with reference to study objectives	L 185-192
Limitations	19	Discuss limitations of the study, taking into account sources of potential bias or imprecision. Discuss both direction and magnitude of any potential bias	L 273-287
Interpretation	20	Give a cautious overall interpretation of results considering objectives, limitations, multiplicity of analyses, results from similar studies, and other relevant evidence	L 252-272
Generalisability	21	Discuss the generalisability (external validity) of the study results	L 363-368
**Other information**			
Funding	22	Give the source of funding and the role of the funders for the present study and, if applicable, for the original study on which the present article is based	L 369-370

*Give information separately for cases and controls.

Note: An Explanation and Elaboration article discusses each checklist item and gives methodological background and published examples of transparent reporting. The STROBE checklist is best used in conjunction with this article (freely available on the Web sites of PLoS Medicine at http://www.plosmedicine.org/, Annals of Internal Medicine at http://www.annals.org/, and Epidemiology at http://www.epidem.com/). Information on the STROBE Initiative is available at http://www.strobe-statement.org.
